# Porcine Reproductive and Respiratory Syndrome Virus Nonstructural Protein 4 Induces Apoptosis Dependent on Its 3C-Like Serine Protease Activity

**DOI:** 10.1371/journal.pone.0069387

**Published:** 2013-07-23

**Authors:** Zhitao Ma, Yalan Wang, Haiyan Zhao, Ao-Tian Xu, Yongqiang Wang, Jun Tang, Wen-hai Feng

**Affiliations:** 1 State Key Laboratory of Agrobiotechnology, China Agricultural University, Beijing, China; 2 Department of Microbiology and Immunology, College of Biological Science, China Agricultural University, Beijing, China; 3 Department of Basic Veterinary Medicine, College of Veterinary Medicine, China Agricultural University, Beijing, China; 4 Key Laboratory of Animal Epidemiology and Zoonosis, Ministry of Agriculture, College of Veterinary Medicine, China Agricultural University, Beijing, China; University of Georgia, United States of America

## Abstract

Porcine reproductive and respiratory syndrome (PRRS) is a highly contagious disease in pigs caused by PRRS virus (PRRSV). Although PRRSV infection-induced cell apoptosis has been established, the related viral protein is still unknown. Here, we reported that PRRSV nonstructural protein 4 (nsp4) was a critical apoptosis inducer. Nsp4 could activate caspase-3, -8, and -9. Using truncated constructs without different domains in nsp4, we demonstrated that the full-length of nsp4 structure was required for its apoptosis-inducing activity. Furthermore, using site-directed mutagenesis to inactivate the 3C-like serine protease activity of nsp4, we showed that nsp4-induced apoptosis was dependent on its serine protease activity. The ability of nsp4 to induce apoptosis was significantly impaired by His39, Asp64, and Ser118 mutations, suggesting that His39, Asp64, and Ser118 were essential for nsp4 to trigger apoptosis. In conclusion, our present work showed that PRRSV nsp4 could induce apoptosis in host cells and might be partially responsible for the apoptosis induced by PRRSV infection. PRRSV 3C-like protease-mediated apoptosis represents the first report in the genus *Arterivirus*, family *Arteriviridae*.

## Introduction

Porcine reproductive and respiratory syndrome (PRRS) is an economically important disease of swine industry worldwide, which is characterized with respiratory illness in piglets and severe reproductive problems in sows and gilts [1,2]. PRRS was first reported in the United States in 1987 and in the Netherlands in 1991 [3]. The etiologic agent, PRRS virus (PRRSV), is an enveloped single-stranded positive sense RNA virus and is classified into the genus *Arterivirus*, family *Arteriviridae*, order *Nidovirales* [4]. The genome is approximately 15.4 kb in length and contains 10 open reading frames (ORFs), designated as ORF1a, ORF1b, and ORFs 2-7. ORFs 2-7 encode structural proteins including GP2a, GP2b, GP3, GP4, GP5, GP5a, M, and N protein, respectively. The replicative enzymes of PRRSV are encoded in ORF1a and ORF1b, which constitute almost 75% of the genome [1]. ORF1a and ORF1b encode polyproteins, pp1a and pp1ab, and expression of pp1ab depends on a ribosomal frameshift signal in the ORF1a/ORF1b overlap region [5]. At least 14 nonstructural proteins (nsp) are generated as a result of serial cleavages of pp1a and pp1ab, including nsp1a, nsp1β，nsp2, nsp2TF, nsp3, nsp4, nsp5, nsp6, nsp7, nsp8，nsp9，nsp10，nsp11，and nsp12 [1,6]. The processing of pp1a and pp1ab is believed to be mediated by accessory proteases, nsp1 and nsp2, and the main serine protease nsp4 [1]. Nsp4 serine protease is responsible for most of the nonstructural protein processing [7,8], and is a member of a relatively rare group of proteolytic enzymes, 3C-like serine proteases named after the picornavirus 3C protease [4,5,9]. The monomeric enzyme, nsp4, folds into three domains including two chymotrypsin-like β-barrel domains and an extra C-terminal α/β domain, which are located at 1-69 amino acids (aa) (domains I), 89-153 aa (domains II), and 157-199 aa (domains III), respectively [8]. The active site of nsp4 is located between domains I and II, and contains a canonical catalytic triad of His39, Asp64, and Ser118 [8].

There are two apoptosis pathways: the extrinsic pathway (death receptor pathway) and the intrinsic pathway (the mitochondria pathway). Caspase-8 and caspase-9 are initiator caspases that mediate the extrinsic and the intrinsic pathways, respectively. Both caspase-8 and caspase-9 can activate caspase-3, a crucial effector caspase conducting the final execution steps of apoptosis program [10–12]. Besides, activated caspase-8 can also cause the cleavage of Bid to yield a truncated form, tBid [11,12]. tBid translocates to mitochondria to induce the release of cytochrome C, and then activate caspase-9, which is the crosstalk between the extrinsic and intrinsic pathways [11].

It has been well established that PRRSV infection leads to apoptosis in infected cells and bystander cells both *in vitro* and *in vivo* [13–19]. Subsequently, a question has been raised: which viral component contributes to the apoptosis-inducing ability of PRRSV. GP5 has been reported to be an apoptosis inducer [20,21]. However, Lee et al [22] demonstrated that cells stably expressing GP5 did not show any characteristics of apoptosis. Thus, the viral proteins related to the PRRSV apoptosis-inducing ability remain unclear and are of huge interests in the field.

In this study, we provided evidence that nsp4 could cause apoptosis in many cell lines. We showed that nsp4 triggered apoptosis through caspase-3, -8, and -9 activations. Using deletion analysis, we demonstrated that all domains of nsp4 were required for it to induce apoptosis. Nsp4-induced cell apoptosis was dependent on its 3C-like serine protease activity, and His39, Asp64, and Ser118 were proved to be essential for nsp4 to trigger apoptosis by point mutagenesis.

## Materials and Methods

### Ethics statement

All animal research was approved by the Beijing Association for Science and Technology (approval ID SYXK (Beijing) 2007-0023) and complied with the guidelines of Beijing Laboratory Animal Welfare and Ethics of the Beijing Administration Committee of Laboratory Animals. All animal studies were also performed in accordance with the China Agricultural University Institutional Animal Care and Use Committee guidelines (ID: SKLAB-B-2010-003) and approved by animal welfare committee of China Agricultural University. All surgery was performed under sodium pentobarbital anesthesia, and all efforts were made to minimize suffering.

### Cells and viruses

Marc-145 cells (ATCC No. CRL-12231), a PRRSV-permissive cell line subcloned from MA-104 cells, were maintained in Dulbecco’s minimum essential medium (DMEM, Gibco, USA) supplemented with 10% FBS and penicillin/streptomycin. Porcine alveolar macrophages (PAMs) were obtained by postmortem lung lavage of 8-week-old specific pathogen free pigs and maintained in RPMI 1640 (Gibco, USA) supplemented with 10% FBS and penicillin/streptomycin. COS-1 cells (ATCC No. CRL-1650) were obtained from cell resource center in Peking Union medical college, and grown in DMEM supplemented with 10% FBS and penicillin/streptomycin. Hela cells were cultured in RPMI 1640 supplemented with 10% FBS and penicillin/streptomycin.

PRRSV strain, CH-1a (the first type 2 PRRSV strain isolated in China), and HV (a highly pathogenic PRRSV (HP–PRRSV) isolate) were propagated in PAMs. Virus preparations were titrated, and then stored at -80°C.

### Plasmids’ construction and transfection

The PRRSV nsp4 expression plasmid was constructed by cloning the coding sequence of nsp4 from HP–PRRSV HV strain into the pcDNA3.1 (+) and pCMV-Myc. Truncated PRRSV nsp4 mutants and point mutants were constructed using specific primers listed in Table 1 and Table 2, and subcloned into the pCMV-Myc. The gene of MID and point mutants (H39A, D64A, S118A) were obtained by overlap extension PCR. All the constructs were validated by DNA sequencing.

**Table 1:** Sequences of the primers used for NSP4 protein deletion mutants.

Name	Sequence
N69 forward	5'-GCGTCGACAATGTGCCCGAATTGGCAAG-3'
N69 reverse^2^	5'-ATTTGCGGCCGCTCATTCCAGTTCAGGTTTGGCA-3'
MID forward-3	5'-TGGGCTTCACAGTCCACC-3'
MID reverse-3	5'-GTGAAGCCCATCAAGCTGAG-3'
C157 forward^1^	5'-GCGTCGACCATGGGTGCTTTCAGAACTCAA-3'
C157 reverse	5'-ATTTGCGGCCGCTCAGGGCTTCACATTACAAAAC-3'

^1^ The forward primer of MID and C157 deletion mutants.

^2^ The reverse primer of N69 and MID deletion mutants.

**Table 2:** Sequences of the primers used for NSP4 protein piont mutants.

Name	Sequence
C157 forward^1^	5'-GCGTCGACCATGGGTGCTTTCAGAACTCAA-3'
H-A reverse-3	5'-CCGTAAGGACTGCTGCGGC-3'
H-A forward-3	5'-GTCCTTACGGGTAACTCAGCTAG-3'
D-A reverse-3	5'-CTATGGCGAATGCCCCTTTTAC-3'
D-A forward-3	5'-TTCGCCATAGCTGATTGCCC-3'
S-A reverse-3	5'-CTGGGGATCCTGCATCGCCACAC-3'
S-A forward-3	5'-GGATCCCCAGTGATTACCGAAG-3'
N69 reverse^2^	5'-ATTTGCGGCCGCTCATTCCAGTTCAGGTTTGGCA-3'

^1^ The forward primer of H39A, D64A and S118A piont mutants.

^2^ The reverse primer of H39A, D64A and S118A piont mutants.

Plasmids were transfected into cells using TurboFect™ *in vitro* Transfection Reagent (ThermoScientific, USA). All transfections were performed following manufacturer’s recommendations.

### Construction, expression, and purification of PRRSV pT7His-nsp4, H39A, D64A, S118A and pET32a-NSP3’4

Genes encoding the wild-type 3C-like protease and the single-point mutant proteases were amplified by PCR from nsp4 and single-point mutant plasmids with the primer pairs as follow: forward primer 5'-ATACATATGCACCATCATCATCATCATCATTCATCAGGTGCTTTCAGAACTCAA-3', and reverse primer 5'-TTGCTCGAGTCATTCCAGTTCAGGTTTG-3'. The PCR products were placed downstream of the T7 promoter with His_6_-tag at N-terminus, and the resultant plasmids were designated as pT7His-nsp4, H39A, D64A, and S118A, respectively. NSP3'4 covered the last 8 residues of nsp3 and the whole amino acid sequence of nsp4 (with Ser118 substituted for Ala). NSP3'4 gene sequence was amplified by PCR from S118A plasmid. The primer pairs were shown as follow: forward primer 5'-CTTGGTACCTCTCAGCTCGGGTCCCTCCTTGAGGGTGCTTTCAGAACTC-3', and reverse primer 5'-CCCAAGCTTTCATTCCAGTTCAGGTTTG-3'. The PCR products were cloned into the pET32a vector, and the constructed vector was designated as pET32a-NSP3’4. All constructs were validated by DNA sequencing.

The expression and purification of PRRSV wild-type and mutant nsp4 proteolytic enzymes and NSP3'4 were performed as described previously [23]. Briefly, the plasmids pT7His-nsp4, H39A, D64A, S118A, and pET32a-NSP3’4 were transformed into *E. Coli* BL21 (DE3). Bacteria were cultured at 37°C until the concentration at A_600_ reached to 0.6-0.8, and then induced with IPTG for 5 h. Ni-NTA column was used to purify the recombinant proteins.

### Proteolytic reaction

Proteolytic reaction was conducted as reported before [23]. The wild-type and mutant nsp4 proteolytic enzymes (5 μM) were reacted with the substrate NSP3'4 (5 μM) in 50 μl of 50 mM Tris/HCl buffer (pH 7.5) containing 100 mM NaCl for 24 h at 8°C. The proteolytic reaction was stopped by adding a quarter volume of 5× sample buffer. The cleaved proteins were analyzed by 17.5% (v/v) sodium dodecyl sulfate-polyacrylamide gel electrophoresis (SDS-PAGE).

### Western blot analysis

Marc-145 cells, Hela cells, or COS-1 cells cultured in 6-well plates were transfected with 4 μg of nsp4, deletion or point mutant plasmids. At 48 h post transfection, cells were washed and then lysed with lysis buffer. The concentrations of proteins in whole cellular lysates were assessed. Same amounts of proteins from each sample were separated by 15% (v/v) SDS-PAGE and transferred to PVDF membranes. After blocking, the membrane was incubated for 1 h at room temperature with the following primary antibodies: nsp4 polyclonal antiserum (1:5,000), anti-myc (1:2,000; MBL), anti-β-actin (1:5,000; Sigma), anti-caspase-3 (1:500; Beyotime), or anti-PARP (1:1,000; Beyotime). The membrane was then incubated with the appropriate secondary antibody for 1 hour. The antibodies were visualized with the ECL reagent according to the manufacturer’s instructions.

### TUNEL assay

Detection of cell apoptosis was performed using the TUNEL technique following the manufacturer’s protocol (Roche). Brieﬂy, cells were ﬁxed with 4% paraformaldehyde in PBS for 1 h at room temperature. After rinsing with PBS, cells were permeabilized using 0.3% Triton X-100 in 0.1% sodium citrate for 4 min. Cells were subsequently stained with the TUNEL reaction mixture for 2 h at 37°C in the dark. After being washed with PBS, cells were counterstained with DAPI. Stained cells were analyzed with a ﬂuorescence microscope. The six visual fields were randomly selected, and FITC-positive and DAPI-positive cells were counted. The amount of DAPI-positive cells were regarded as the total cells number. Results were expressed as percentage of FITC-positive cells.

### Annexin V / PI staining assay

Cell apoptosis rates were analyzed by staining cells with Annexin V-FITC Apoptosis Detection Kit (BD Biosciences) following the manufacturer’s instructions. After staining, data on 20,000 cells were acquired using a FACS Calibur (BD Bioscience) and analyzed by FlowJo software. Results were expressed as percentages of the FITC-positive and PI-negative cells.

### Double-labeling immunofluorescence assay

Cells grown on cover slips were fixed in 4% paraformaldehyde in PBS for 1 h at room temperature. TUNEL assays were carried out first as described above. And then, indirect immunofluorescence staining was performed as follow. Cells were washed, blocked, and then incubated for 2 h at 37°C with the primary antibodies: mouse monoclonal antibody against PRRSV N protein SDOW17 (1:10,000; Rural Technologies) or anti-myc mouse monoclonal antibody (1:1,000; MBL). After washing, cells were incubated for 2 h at 37°C with the anti-mouse lgG antibody conjugated with TRITC (1:100; Sigma). After three washes in PBS, cells were counter-stained with DAPI and examined by fluorescence microscopy.

### Analysis of caspase enzymatic activity

PAMs were infected with PRRSV (HV strain) at an MOI of 0.5. Hela cells were transfected with pCMV-Myc control and nsp4, respectively. Twenty-four hours post infection or 48 h post transfection, the enzymatic activities of caspase-3, -8, and -9 were examined using colorimetric assays following the manufacturer’s protocol (Beyotime, China). Brieﬂy, 10^6^ cells were harvested, lysed, and centrifuged at 12,000 g for 15 min at 4°C. The concentrations of proteins in the supernatant were assessed with a BCA protein assay kit (Beyotime). A 20 μg aliquot of proteins was incubated with the colorimetric substrate DEVD-pNA for the caspase-3 assay, IETD-pNA for the caspase-8 assay, and LEHD-pNA for the caspase-9 assay for 2 h at 37°C. Photometric analysis was performed at 405 nm, and background values obtained from wells without colorimetric substrate were subtracted. The fold increases of caspase activity in infected or transfected cells were quantitated relative to the control cells.

### Statistical analysis

All experiments were performed with at least three independent replicates. Results were analyzed using GraphPad Prism software, and differences were evaluated by *Student’s t test* (paired). *P* values of less than 0.05 were considered statistically signiﬁcant. *P<0.05, ** *P<0.01*, ***P<0.001. 

## Results

### PRRSV infection induces apoptosis in PAMs and Marc-145 cells

**Figure 1:**
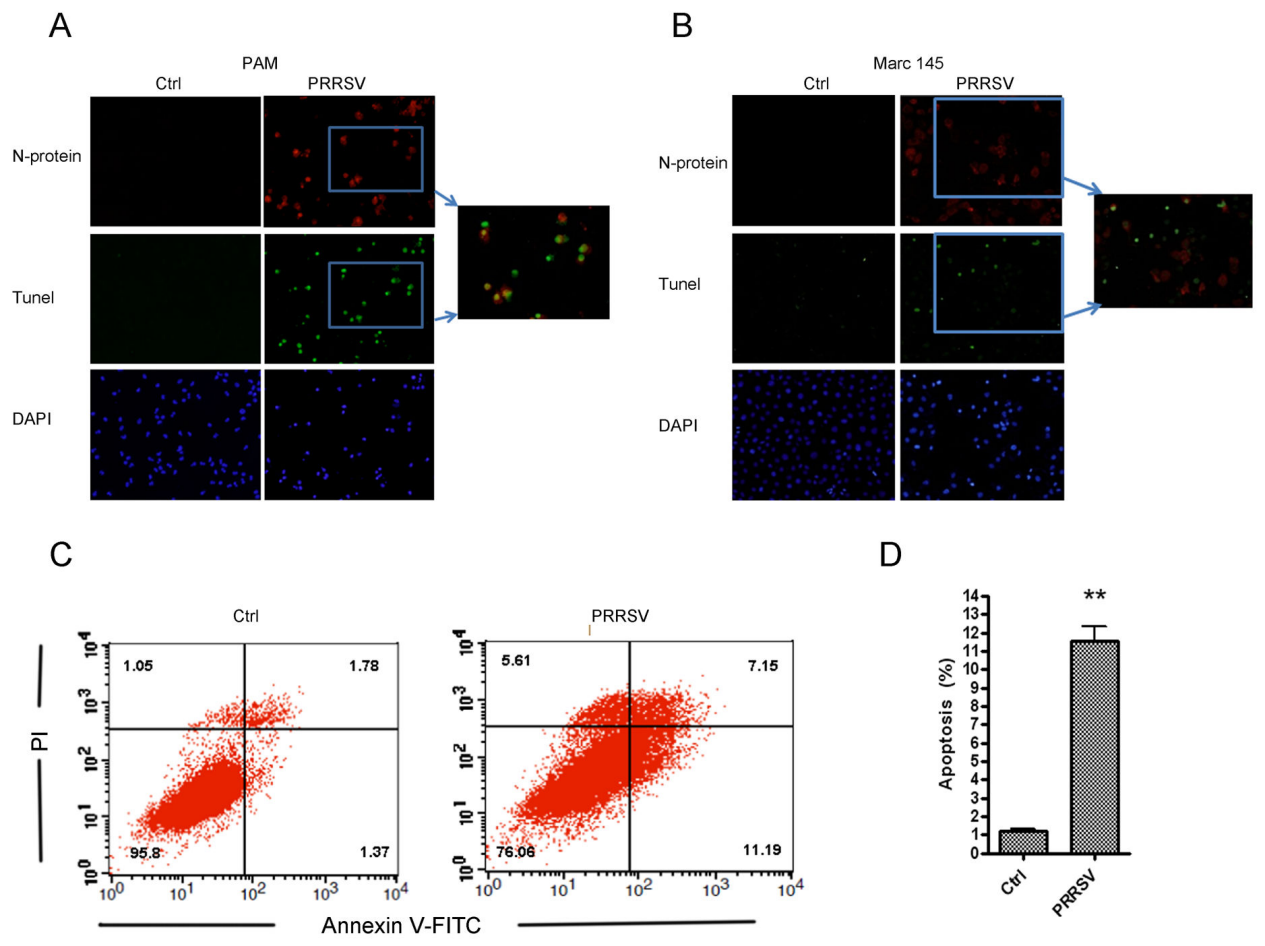
PRRSV infection induced apoptosis in PAMs and Marc-145 cells. (A and B) Immunofluorescence analysis of apoptosis in PAMs and Marc-145 cells. Apoptosis (green), PRRSV N protein (red), and nucleus (blue) were detected. (A) PAMs were mock infected or infected with HP–PRRSV (HV strain) at an MOI of 0.5. At 24 hours post infection, cells were fixed, stained, and observed by ﬂuorescence microscopy. (B) Marc-145 cells were mock infected or infected with PRRSV (CH-1a strain) at an MOI of 1. At 48 hours post infection, cells were fixed, stained, and observed by ﬂuorescence microscopy. (C) Flow cytometry analysis of apoptosis in Marc-145 cells at 48 hours after infection with PRRSV (CH-1a strain) at an MOI of 1.0. Cells were collected, stained with Annexin V/PI, and analyzed by flow cytometry. Mock-infected cells were used as controls. (D) Percentage of apoptotic cells in panel C. Graphs show means ± SD of three independent experiments. *P<0.01* (**) as determined by *student’s t test*.

PRRSV has been found to be capable of inducing apoptosis in PAMs and Marc-145 cells [24]. To confirm this, we infected PAMs with HP–PRRSV HV strain at an MOI of 0.5 and then examined apoptosis using TUNEL assay at 24 h post infection. Our results showed that the number of apoptotic cells increased significantly after PRRSV infection in PAMs (Figure 1A). PRRSV-induced apoptosis was also evaluated in Marc-145 cells. As shown in Figure 1B, more apoptotic cells were observed in Marc-145 cells infected with PRRSV CH-1a strain when compared to that in mock infected cells. And analysis by flow cytometry showed that the percentage of apoptotic Marc-145 cells infected with PRRSV was remarkably higher when compared to that of mock-infection (11.54 ± 0.84% versus 1.21 ± 0.13%) (Figure 1C and 1D).

### Nsp2 and nsp4 induce apoptosis in Marc-145 cells

**Figure 2:**
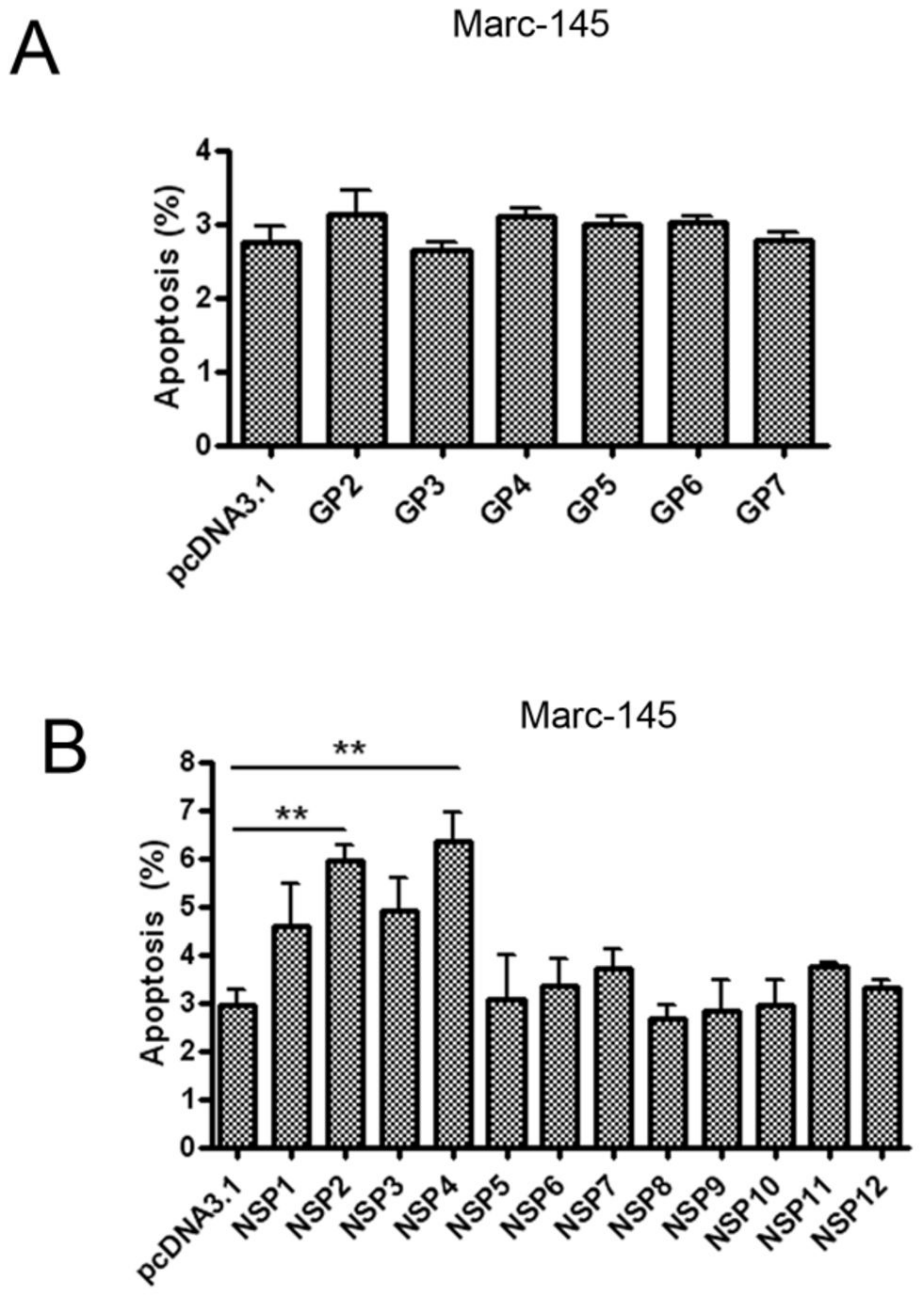
PRRSV nonstructural proteins nsp2 and nsp4 induced apoptosis. Marc-145 cells were transfected with each of the PRRSV structural protein expression vectors (A), nonstructural protein expression vectors (B), or pcDNA 3.1 (+) vector as control. At 48 hours post transfection, cells were fixed for TUNEL analysis. Results represent means ± SD of three independent experiments. *P<0.01* (**) as determined by *student’s t test*.

Given that PRRSV infection can cause apoptosis in PAMs and Marc-145 cells, we then sought to determine which viral gene(s) could induce host cell apoptosis. Marc-145 cells were transfected with each of the PRRSV structural and nonstructural protein expression vectors. Our results showed that none of the PRRSV structural proteins had the potential to cause apoptosis (Figure 2A). However, we found that both nsp2 and nsp4 could induce apoptosis in Marc-145 cells with apoptosis rates of 6.35 ± 0.6% for NSP4 and 5.96 ± 0.32% for NSP2 (Figure 2B).

### Nsp4 could induce apoptosis in various cell lines

**Figure 3:**
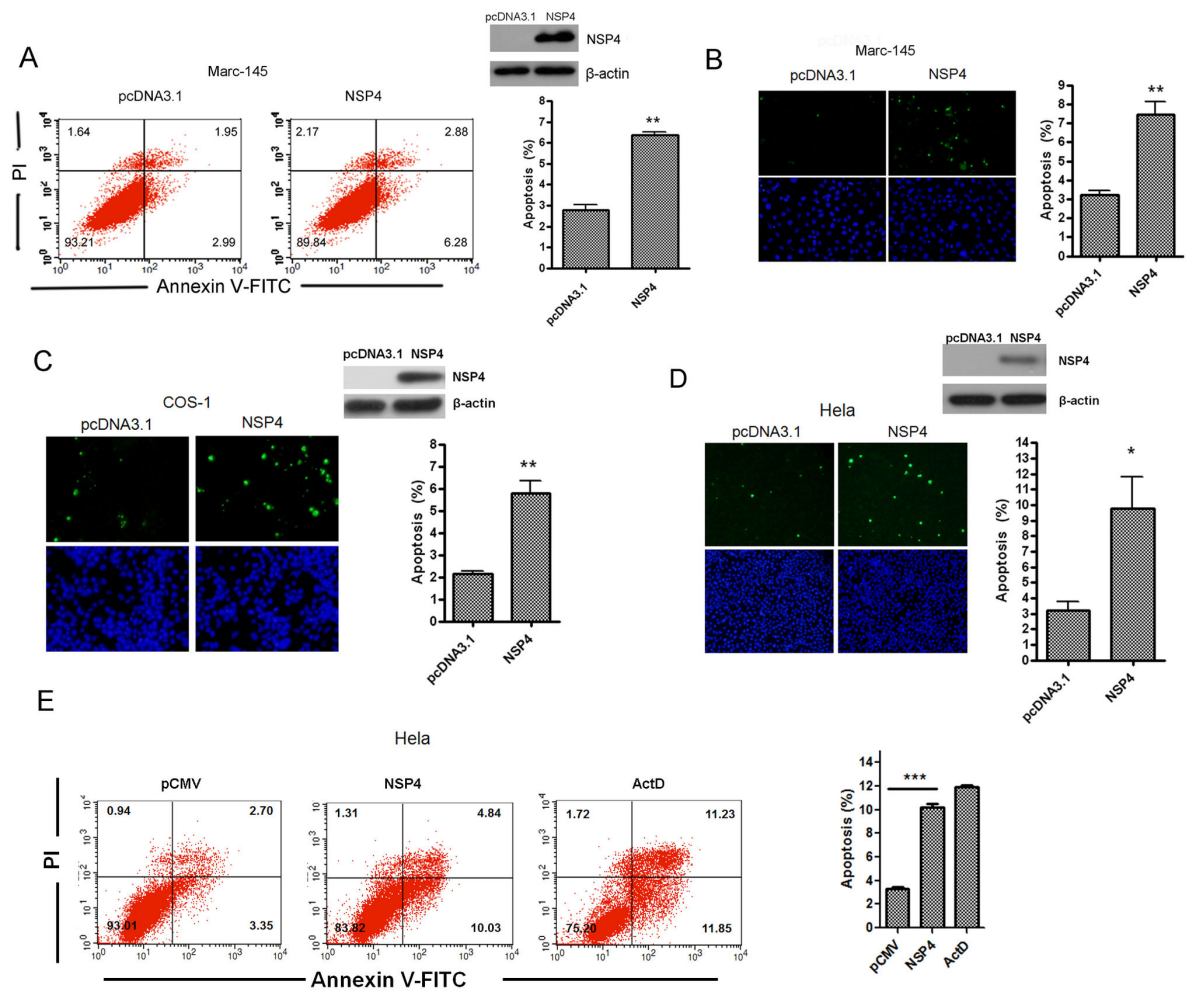
PRRSV nsp4 caused apoptosis in various cell lines. (A) Flow cytometry analysis of nsp4-induced apoptosis in Marc-145 cells. Cells were transfected with nsp4 expression plasmid or pcDNA3.1 (+) as control. At 48 hours post transfection, cells were collected, stained, and analyzed by flow cytometry. (B, C, and D) *In situ* TUNEL analysis of nsp4-induced apoptosis in Marc-145, COS-1, and Hela cells. Marc-145 (B), COS-1 (C), and Hela cells (D) were transfected with either pcDNA 3.1 (+) vector or nsp4 expression vector. Forty-eight hours later, cells were fixed, stained with TUNEL reaction mixture, and then detected by ﬂuorescence microscopy. The expression of nsp4 was examined with western blotting using anti-nsp4 serum. (E) Flow cytometry analysis of nsp4-induced apoptosis in Hela cells. Hela cells were transfected with pCMV-Myc control or nsp4-expressing plasmid, or treated with Actinomycin D (ActD) at a concentration of 15 ng/ml as a positive control, respectively. After 48 hours, cells were collected, stained, and analyzed by flow cytometry. Results represent means ± SD of three independent experiments. *P<0.01* (**) *P<0.001* (***) as determined by *student’s t test*.

Since nsp2 accounts for the major genetic differences between PRRSV strains and nsp4 is highly conserved among PRRSV strains [1,5,7,25], we then focused on nsp4. To further confirm that nsp4 could induce apoptosis, we transfected Marc-145 cells with nsp4. As shown in Figure 3A and Figure 3B, the apoptotic percentage of nsp4-transfected cells was approximately 2.3 times higher than that of control (8.93±0.81% versus 3.87±0.27%). We also examined its potential to induce apoptosis in other cell lines. As expected, nsp4 induced apoptosis in COS-1 cells and Hela cells (Figure 3C, 3D and 3E). Besides, Nsp4 protein also caused apoptosis in mouse neuroblastoma N2a cells (data not show).

### PRRSV and nsp4 activate caspase-3, -8 and -9

**Figure 4:**
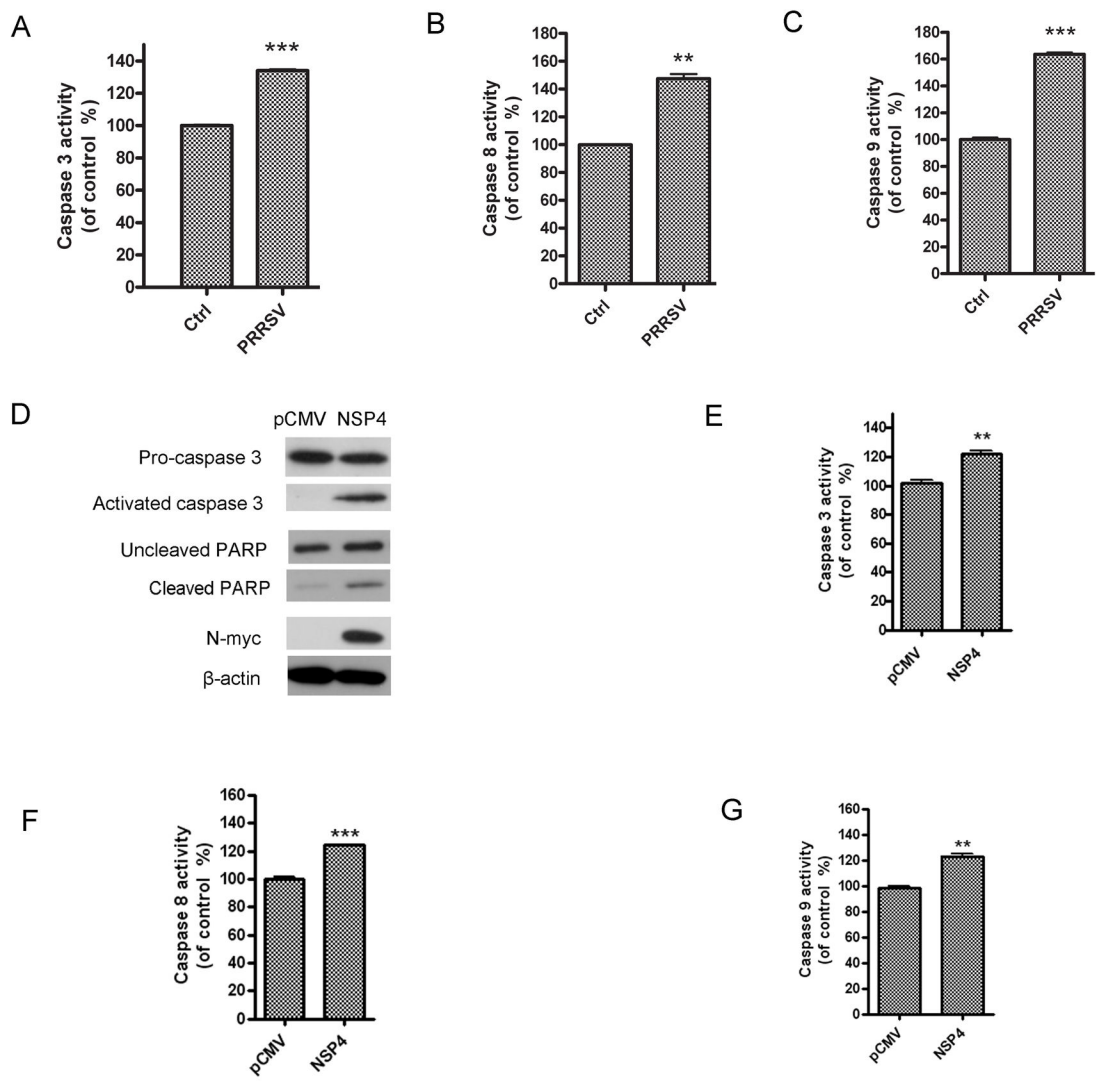
PRRSV-infection and nsp4-transfection activated caspases. (A, B, and C) PAMs were mock infected or infected with HP–PRRSV (HV strain) at an MOI of 0.5. At 24 hours post infection, the enzymatic activities of caspase-3, -8, and -9 were examined using colorimetric assays. (D) Hela cells were transfected with pCMV-Myc control or nsp4-expressing plasmid. At 48 hours post transfection, activated caspase 3 (17 kDa), cleaved PARP, and nsp4 were analyzed using western blot. (E, F and G) Hela cells were transfected with pCMV-Myc control or nsp4-expressing plasmid. At 48 hours post transfection, the enzymatic activities of caspase-3, -8, and -9 were examined using colorimetric assays. Data represent means ± SD of three independent experiments. *P<0.05* (*), *P<0.01* (**), *P<0.001* (***) as determined by *student’s t test*.

PRRSV has been reported to induce apoptosis through the caspase-mediated pathway [24]. To further confirm the effect of PRRSV infection on the apoptosis pathways, we examined the activities of caspase-3, -8, and -9 in PAMs infected with HP–PRRSV HV strain with colorimetric assays. Our results showed that caspase-3, -8 and -9 activities in PRRSV-infected PAMs were significantly higher than that in control cells (Figure 4A, B and C). These data suggested that caspase-3, -8, and -9 activation was involved in the induction of apoptosis by PRRSV infection. Subsequently, we tested if nsp4 could activate caspase family using western blot analysis. As shown in Figure 4D, cleaved PARP (about 89kD) as well as the activated caspase-3 (about 17kD) was observed in nsp4-transfected cells, indicating that caspase-3 was activated. We further assessed caspase-3, -8 and -9 activities using colorimetric assays, and observed a similar increase in their activities in nsp4-transfected cells (Figure 4E, F and G).

### Full-length sequence is required for nsp4 to induce apoptosis

**Figure 5:**
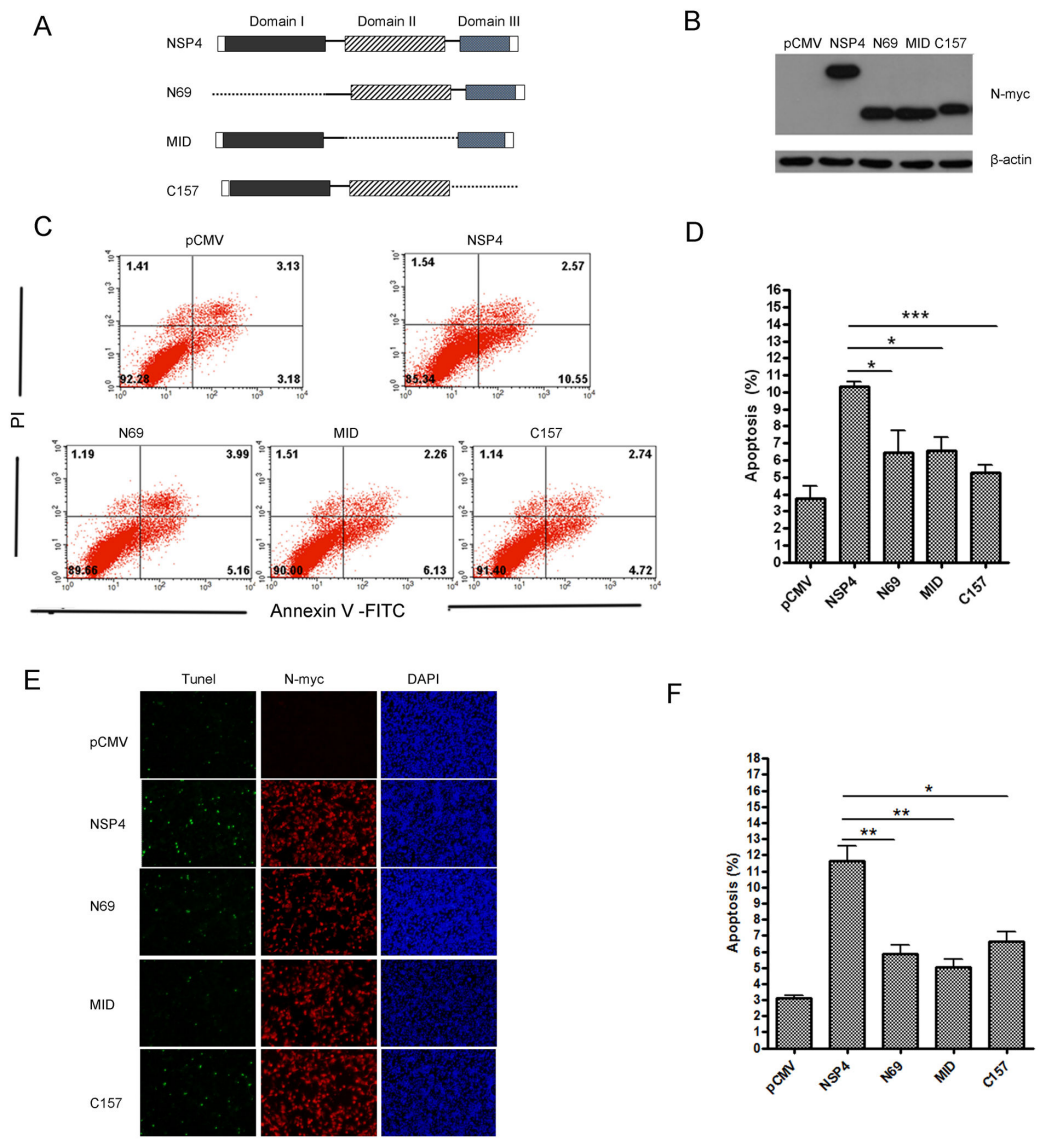
Deletion of either of the three PRRSV nsp4 domains impaired its ability to induce apoptosis. (A) Schematic diagram represents the PRRSV nsp4 protein deletion mutant constructs. (B) Expression of nsp4, N69, MID, and C157 proteins in Hela cells. Cells were transfected with pCMV-Myc, wild-type nsp4, or deletion mutant plasmids. At 48 hours post transfection, cells were lysed for western blotting to verify proteins expression using anti-myc antibodies. (C) Flow cytometry analysis of wild-type nsp4 and deletion mutants-induced apoptosis in Hela cells at 48 hours post transfection. (D) Percentage of apoptotic cells in panel C. (E) Double-labeling immunofluorescence analysis using TUNEL for apoptosis and indirect immunofluorescence for wild-type and mutant nsp4 proteins in Hela cells at 48 hours post transfection. Apoptosis (green), wild-type or mutant nsp4 (red), and nucleus (blue) were detected by immunofluorescence staining. (F) Percentage of apoptotic cells in panel E. Data represent means ± SD of three independent experiments. *P<0.05* (*), *P<0.01* (**), *P<0.001* (***) as determined by *student’s t test*.

PRRSV nsp4 includes three domains located at amino acids 1-69,89-153, and 157-199, respectively [8]. To investigate which domain is responsible for apoptosis induction, we deleted each of the three domains and designated the mutants as N69 (1~69aa deleted), MID (89~153aa deleted), and C157 (157~204aa deleted), respectively. The three mutant constructs were shown schematically in Figure 5A, and their expressions were confirmed by western blot analysis (Figure 5B). Next, we investigated the abilities of the deletion mutants to induce apoptosis in Hela cells. As shown in Figure 5C and Figure 5D, the apoptosis level declined remarkably in N69, MID, and C157-transfected cells compared to that in wide-type nsp4-transfected cells. In addition, with the double-labeling assay, we observed that wild-type and mutant nsp4 proteins were expressed with no significant differences (Figure 5E). However, apoptosis-inducing abilities of N69, MID, and C157 were nearly abolished when compared to wild-type nsp4 (Figure 5F). Taken together, these data suggest that all the three domains are critical for nsp4 to induce apoptosis.

### His39, Asp64, and Ser118 are essential for nsp4 to induce apoptosis, and nsp4-induced apoptosis is dependent on its 3C-like serine protease catalytic activity

**Figure 6:**
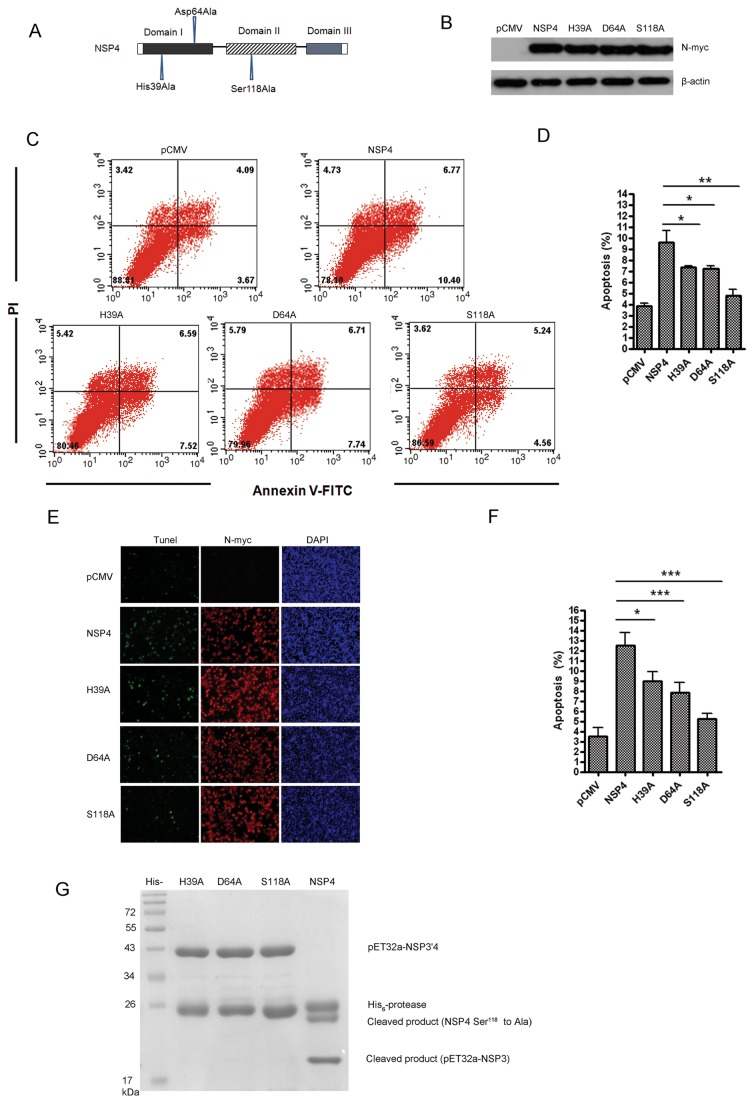
His39, Asp64, and Ser118 were required for nsp4 to induce apoptosis, and nsp4-induced apoptosis was dependent on its 3C-like serine protease activity. (A) Schematic diagram of the PRRSV nsp4 protein and point mutant constructs. (B) Expression of nsp4, H39A, D64A, and S118A point mutant proteins in Hela cells. Cells were transfected with wild-type nsp4, H39A, D64A, or S118A point mutant plasmid. At 48 hours post transfection, cell lysates were prepared and examined with western blotting. (C) Flow cytometry analysis of nsp4, H39A, D64A, or S118A-induced apoptosis in Hela cells at 48 hours post transfection. (D) Percentage of apoptotic cells in panel C. Data are representatives of three independent experiments. (E) Double-labeling immunofluorescence analysis using TUNEL for apoptosis and indirect immunofluorescence for wild-type and the point mutant nsp4 proteins in Hela cells at 48 hours post transfection. Apoptosis (green), wild-type or mutant nsp4 (red), and nucleus (blue) were detected. (F) Percentage of apoptotic cells in panel E. (G) Proteolytic reaction was mediated by PRRSV wild-type and point mutant nsp4 proteins. The wild-type or point mutant nsp4 proteins were incubated with the substrate NSP3^’^ 4 (Ser118Ala) for 24 h. Lanes 2 through 5, H39A, D64A, S118A, and wild-type nsp4. Graphs show means ± SD from three independent experiments. *P<0.05* (*), *P<0.01* (**), *P<0.001* (***) as determined by *student’s t test*.

Truncation of proteins may compromise nsp4 overall structure. Thus, more subtle changes were introduced to map residues involved in its apoptosis-inducing functions. Nsp4 is a 3C-like serine protease that contains the canonical catalytic triad of His39–Asp64–Ser118. To assess the role of 3C-like serine protease activity in nsp4-triggered apoptosis, three single-point mutants were generated, including His39Ala (H39A), Asp64Ala (D64A), and Ser118Ala (S118A). The mutants were schematically shown in Figure 6A, and the protein expression of each mutant was confirmed using western blot analysis (Figure 6B). Subsequently, the apoptosis rates induced by each mutant were analyzed by flow cytometry using Annexin V/PI staining in Hela cells. As shown in Figure 6C and Figure 6D, the apoptosis rate induced by either H39A, D64A, or S118A mutant was significantly lower compared to that of wild-type nsp4 with a reduction of about 23.1%, 24.5%, and 50.1%, respectively. Double-labeling assay showed similar results (Figure 6E and Figure 6F), implying that His39, Asp64, and Ser118 were critical for nsp4 to induce apoptosis.

We next sought to further investigate if nsp4 3C-like protease activity was affected in H39A, D64A, and S118A mutants. The genes encoding wild-type and mutant 3C-like proteases were cloned and expressed with His_6_-tag at N-terminus. NSP3'4 mutant protein was used as the substrate, which included the last 8 residues of nsp3 and the whole amino acid sequence of nsp4 with Ala substitution for Ser118. If the substrate had been cleaved at the nsp3 and nsp4 junction (E/G) by the active 3C-like protease, nsp3 moiety and nsp4 (Ser118 to Ala) moiety would be expected. As shown in Figure 6G, although NSP3'4 had the 3C-like protease sequence, autocatalytic cleavage did not occur because of the Ser118 to Ala mutation. Furthermore, the substrate was recognized and cleaved by wild-type nsp4, but not by any of the three nsp4 mutants (Figure 6G, lane 2, 3, 4, and 5). The mutation of His39, Asp64, or Ser118 to Ala caused a complete abolishment of nsp4 cleavage activity, demonstrating that His39, Asp64, and Ser118 were essential for nsp4 protease function.

Collectively, these data suggested that His39, Asp64, and Ser118 were critical for nsp4 to induce cell apoptosis, and nsp4-induced cell apoptosis was dependent on its 3C-like serine protease catalytic activity.

## Discussion

In this study, we investigated the ability of PRRSV proteins to induce apoptosis. By transfecting viral protein genes into cells, we demonstrated that PRRSV nsp4 could induce apoptosis in various cell lines including Marc-145 cells, Hela cells, and COS-1 cells. Nsp4 could activate caspase family, which is in coincident with that of PRRSV infection. Using deletion analysis, we demonstrated that all of the three domains of nsp4 were required for nsp4 to induce apoptosis. We further demonstrated that nsp4-induced cell apoptosis was dependent on its 3C-like serine protease activity, and His39, Asp64, and Ser118 were essential for nsp4 to induce apoptosis.

Apoptosis has been proposed as an immediate host defense response upon viral infections, or serves as a viral strategy to induce cytopathogenicity or to maximize virus progeny. Like many other positive-strand RNA viruses, PRRSV-infection can induce cell apoptosis both *in vivo* and *in vitro* [26]. Evidence of apoptosis has been reported in alveolar macrophages, porcine intravascular monocytes, and lymphocytes in the lungs and lymph nodes of infected pigs, which might be responsible for the dramatic reduction in the number of these cells in PRRSV-infected pigs [19,26,27]. Furthermore, PRRSV could replicate in testicular germ cells and induce germ cell apoptosis *in vivo* [27]. Recently, apoptotic cells were found in B- and T-cell areas of lymphoid organs of infected pigs, as well as in fetal implantation sites at the late stage of gestation [28,29]. In agreement with these observations, we showed that PAMs infected with HP–PRRSV HV strain exhibited characteristic of apoptosis. In addition, apoptosis was detected both in PRRSV-infected and the neighboring cells, which was consistent with the results published before. Apoptosis in uninfected bystander cells could be induced by the local release of cytokines such as TNF-α from infected cells [13,27]. An alternative mechanism could be that increased surface expression FasL on surrounding cells is induced by PRRSV infection, and these cells become sensitive to Fas-mediated apoptosis [24].

GP5 had been reported to be responsible for apoptosis induced by PRRSV infection and the related domain was mapped to the first 119 amino acids subsequently by the same investigators [20,21]. However, in our study we did not observe the apoptosis induced by GP5, which is consistent with the report by Lee et al [22]. Lee et al also showed that GP5 could not induce apoptosis, and speculated that the difference might be due to histidine tag and an antigenic tag placed in the N-terminal of GP5 fusion constructs in previous study, which could disrupt the signal function of the GP5 protein, alter intracellular trafficking of the modified protein, and thus cause apoptosis.

It has been reported that 3C protease of viruses in family *Picornaviridae* (including coxsackievirus B3 (CVB3), enterovirus 71 (EV71), and poliovirus) could cause apoptosis [30–34]. In addition, the role of 3C-like protease in virus-induced apoptosis was also characterized in severe acute respiratory syndrome-associated coronavirus (SARS-CoV) [35]. In the current study, we presented evidence that PRRSV 3C-like protease nsp4 could induce apoptosis in Marc-145 cells and many other cell lines. Interestingly, apoptosis occurred both in nsp4-expressing cells and the surrounding cells, which is similar to the apoptotic phenomenon induced by PRRSV infection. The possibility that nsp4 induces the local release of cytokines (i.e., TNF-α), or the high expression of surface TNF receptor superfamily could not be excluded. Further investigation is required to explain the molecular mechanisms under this phenomenon. Of note, PRRSV nsp4-induced apoptosis represents the first report of 3C-like protease-mediated apoptosis in the genus *Arterivirus*, family *Arteriviridae*.

Apoptosis can be initiated by extrinsic and intrinsic pathways characterized by caspase-8 and caspase-9 activation, respectively. EAV induced apoptosis initiated by caspase-8 activation and subsequently by mitochondria-dependent caspase-9 activation [36]. Similarly, in PRRSV infection, both death receptor and mitochondrial pathways were involved in PRRSV-induced apoptosis, and the potential crosstalk between extrinsic and intrinsic pathways was proven by the dependency of caspase-9 activation on caspase-8 activity and the cleavage of Bid in PRRSV-infected cells [24]. In our study, we showed that caspase-3, -8, and -9 were activated evidently both in PRRSV-infected PAMs and nsp4-transfected cells. Blocking caspase-8 activation by its inhibitor led to a nearly complete inhibition of PRRSV-induced or nsp4-induced apoptosis (data not show), implicating that the crosstalk between intrinsic and extrinsic pathways may exist in nsp4-induced cell apoptosis. Further study is necessary to uncover the mechanisms.

PRRSV nsp4 protein is a 22.4 kDa protein, which consists of three domains. Domain I and II form the typical chymotrypsin-like two-β-barrel fold. The C-terminal domain III, which is dispensable for proteolytic activity, may be involved in polyprotein proteolysis [5]. Using deletion analysis, we showed that each of the three domains was essential for nsp4 to induce cell apoptosis. We also demonstrated that when the 3C-like protease activity of nsp4 was abolished by single-point mutation, the ability of nsp4 to induce apoptosis was significantly impaired. These results were in accordance with the previous report that protease activity was necessary for 3C protease of enterovirus 71 to induce apoptosis in human neural cells [31]. Furthermore, apoptosis was nearly absent in S118A-transfected cells, suggesting that Ser118 might be a more important factor for nsp4 to induce apoptosis. However, we need to indicate that separation of domains might destabilize the protein tertiary structure, resulting in loss of function. So it is hard to conclude the involvement of a particular domain of nsp4 in apoptosis. For the point mutation, even though the effect might be less on the structure, we also could not exclude the possibility that single mutation has effect on its structure, and subsequently lead to the loss of its protease activity.

In conclusion, our results demonstrated that nsp4 could induce cell apoptosis and all the three domains of nsp4 were essential for it to induce apoptosis. We further showed that His39, Asp64, and Ser118 were critical for nsp4 to cause apoptosis. In addition, the ability of nsp4 to trigger apoptosis was proved to be dependent on its 3C-like protease activity. More work focusing on the molecular mechanism underlining PRRSV-induced apoptosis will help us to understand PRRSV pathogenesis.
