# Correction: Porcine Reproductive and Respiratory Syndrome Virus Nonstructural Protein 4 Induces Apoptosis Dependent on Its 3C-Like Serine Protease Activity

**DOI:** 10.1371/journal.pone.0230086

**Published:** 2020-02-28

**Authors:** Zhitao Ma, Yalan Wang, Haiyan Zhao, Ao-Tian Xu, Yongqiang Wang, Jun Tang, Wen-hai Feng

After this article [1] was published, concerns were raised about similarities between flow cytometry results reported in the following figure panels:

- pCDNA3.1 and NSP4 panels in Fig 3A
- MID and C157 panels in Fig 5C

**Fig 3:**
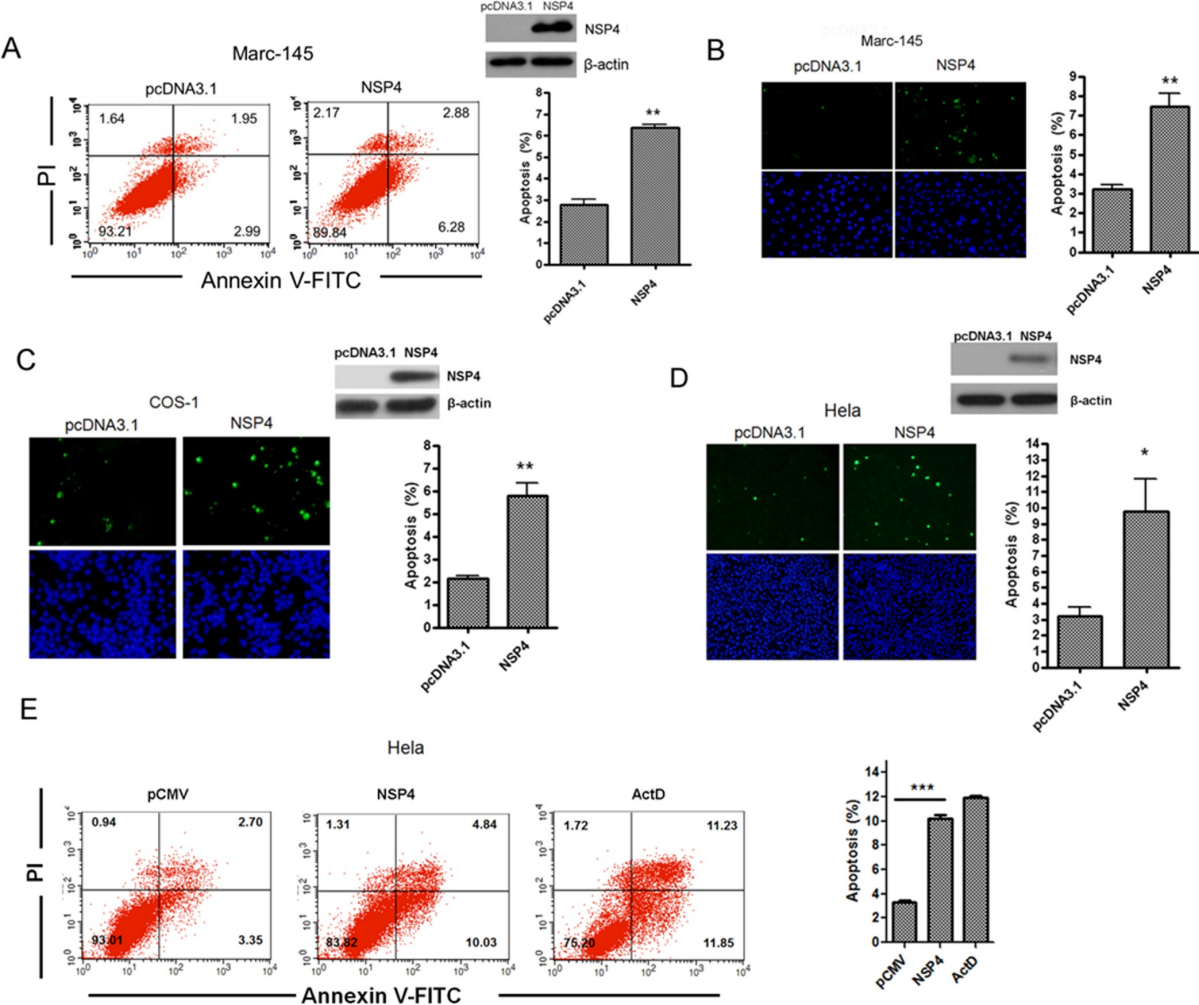
PRRSV nsp4 caused apoptosis in various cell lines. (A) Flow cytometry analysis of nsp4-induced apoptosis in Marc-145 cells. Cells were transfected with nsp4 expression plasmid or pcDNA3.1 (+) as control. At 48 hours post transfection, cells were collected, stained, and analyzed by flow cytometry. (B, C, and D) *In situ* TUNEL analysis of nsp4-induced apoptosis in Marc-145, COS-1, and Hela cells. Marc-145 (B), COS-1 (C), and Hela cells (D) were transfected with either pcDNA 3.1 (+) vector or nsp4 expression vector. Forty-eight hours later, cells were fixed, stained with TUNEL reaction mixture, and then detected by fluorescence microscopy. The expression of nsp4 was examined with western blotting using anti-nsp4 serum. (E) Flow cytometry analysis of nsp4-induced apoptosis in Hela cells. Hela cells were transfected with pCMV-Myc control or nsp4-expressing plasmid, or treated with Actinomycin D (ActD) at a concentration of 15 ng/ml as a positive control, respectively. After 48 hours, cells were collected, stained, and analyzed by flow cytometry. Results represent means ± SD of three independent experiments. *P<0*.*01* (**) *P<0*.*001* (***) as determined by *student’s t test*.

**Fig 5:**
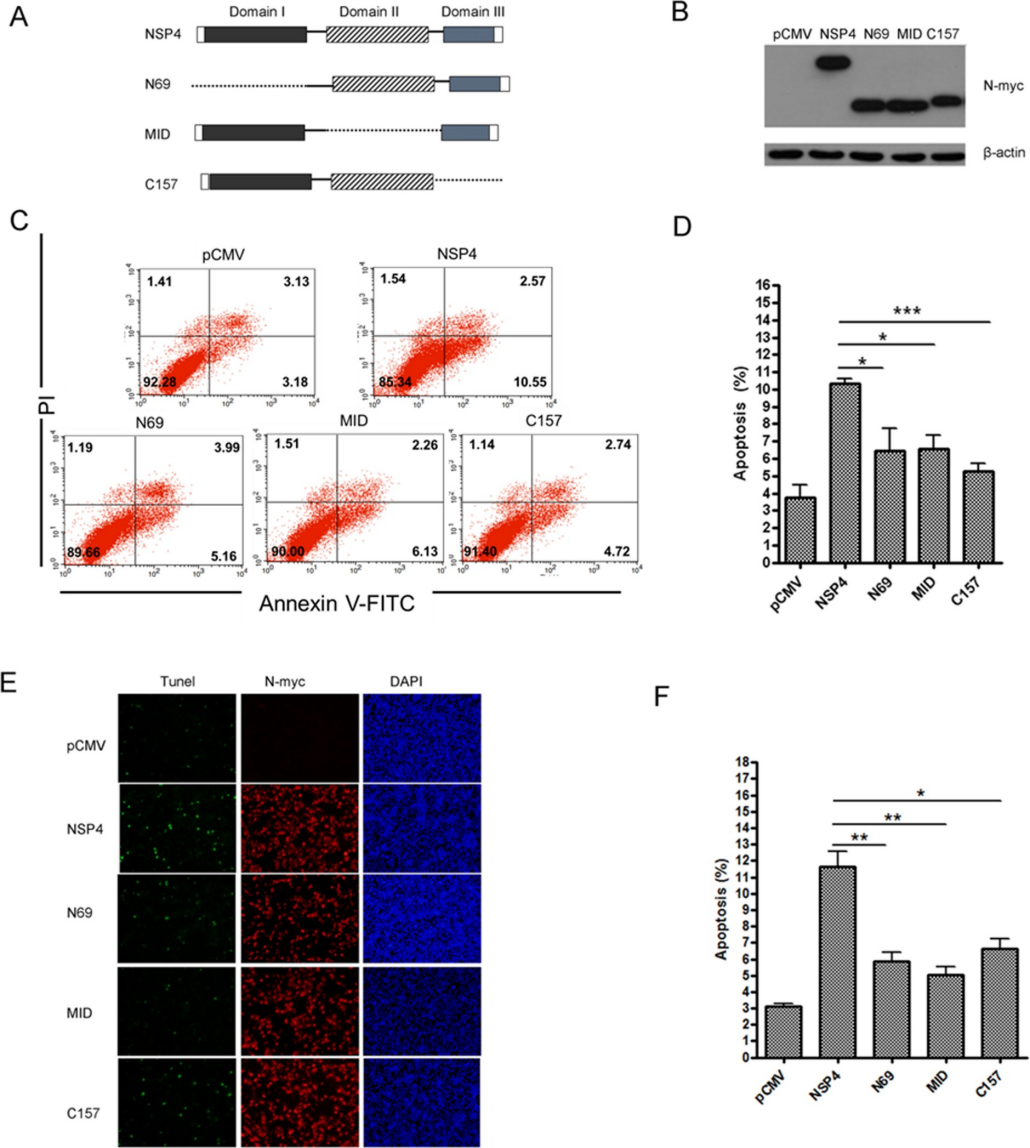
Deletion of either of the three PRRSV nsp4 domains impaired its ability to induce apoptosis. (A) Schematic diagram represents the PRRSV nsp4 protein deletion mutant constructs. (B) Expression of nsp4, N69, MID, and C157 proteins in Hela cells. Cells were transfected with pCMV-Myc, wild-type nsp4, or deletion mutant plasmids. At 48 hours post transfection, cells were lysed for western blotting to verify proteins expression using anti-myc antibodies. (C) Flow cytometry analysis of wild-type nsp4 and deletion mutants-induced apoptosis in Hela cells at 48 hours post transfection. (D) Percentage of apoptotic cells in panel C. (E) Double-labeling immunofluorescence analysis using TUNEL for apoptosis and indirect immunofluorescence for wild-type and mutant nsp4 proteins in Hela cells at 48 hours post transfection. Apoptosis (green), wild-type or mutant nsp4 (red), and nucleus (blue) were detected by immunofluorescence staining. (F) Percentage of apoptotic cells in panel E. Data represent means ± SD of three independent experiments. *P<0*.*05* (*), *P<0*.*01* (**), *P<0*.*001* (***) as determined by *student’s t test*.

The authors noted that due to figure preparation errors the pCDNA3.1 and MID dot plots were mistakenly duplicated in the NSP4 and C157 panels, respectively. These errors are addressed in the revised Figs 3 and 5 provided here, in which the NSP4 (Fig 3A) and C157 (Fig 5C) panels have been replaced with the correct plots from the original experiments. The underlying data for Figs 3A and 5C are no longer available.

A member of *PLOS ONE*’s Editorial Board advised that the updated figures support the results as reported in the original article.

The original data are available upon request from the corresponding author for Figs 1B, 3B, 3C, 4A, 4E, 5E, 6B, 6E. The original underlying data to support the other results in the article are not available, although the authors can provide supporting data for Figs 1A, 2A and 3D that were obtained in replication experiments.

The authors apologize for the errors in the published figures.

## References

[1] Ma Z, Wang Y, Zhao H, Xu A-T, Wang Y, Tang J, et al (2013) Porcine Reproductive and Respiratory Syndrome Virus Nonstructural Protein 4 Induces Apoptosis Dependent on Its 3C-Like Serine Protease Activity. PLoS ONE 8(7): e69387 10.1371/journal.pone.0069387 23936003 PMC3720278

