# Correction: Porcine Reproductive and Respiratory Syndrome Virus Nonstructural Protein 4 Induces Apoptosis Dependent on Its 3C-Like Serine Protease Activity

**DOI:** 10.1371/journal.pone.0356568

**Published:** 2026-08-20

**Authors:** Zhitao Ma, Yalan Wang, Haiyan Zhao, Ao-Tian Xu, Yongqiang Wang, Jun Tang, Wen-hai Feng

After this article [1,2] was published, concerns were raised regarding Figs 5 and 6. Specifically, the pCMV Tunel, N-myc and DAPI panels in Fig 5E appear similar to the pCMV Tunel, N-myc and DAPI panels respectively in Fig 6E.

Co-corresponding author WHF stated that the pCMV Tunel, N-myc and DAPI panels in Fig 5E are incorrect and provided a corrected Fig 5E with the correct panels from the original experiments, provided here as S1 File. The original images underlying all panels in Figs 5E and 6E are provided with this notice as S2 File.

## Supporting information

S1 File Corrected Fig 5E. (TIF)

S2 File Original images underlying all panels in Figs 5E and 6E. (ZIP)
